# Supplementary material for: Discovery and predictive modeling of urine microbiome, metabolite and cytokine biomarkers in hospitalized patients with community acquired pneumonia
Source: Sci Rep. 2020 Aug 7;10:13418. doi: 10.1038/s41598-020-70461-9 (PMC7414893; doi:10.1038/s41598-020-70461-9)
Supplement: Supplementary file 1 — Supplementary Information. [file 41598_2020_70461_MOESM1_ESM.pdf]

Supplemental Figures and Tables

Discovery and Predictive Modeling of Urine Microbiome, Metabolite and Cytokine Biomarkers in Hospitalized Patients with Community Acquired Pneumonia

Joseph F. Pierre[1,3*], Oguz Akbilgic[2*], Heather Smallwood[1*], Xueyuan Cao[5],
Elizabeth A. Fitzpatrick[3], Senen Pena[4], Stephen P. Furmanek[4], Julio A. Ramirez[4], Colleen B Jonsson[3]

[1]*Department of Pediatrics, College of Medicine, University of Tennessee Health Science Center (UTHSC), Memphis, TN*

[2]Department of Health Informatics and Data Sciences, Parkinson School of Health Informatics and Public health, Loyola University Chicago, Maywood, IL, 60153

[3]*Department of Microbiology, Immunology, & Biochemistry, College of Medicine, UTHSC*

[4]*Division of Infectious Diseases, School of Medicine, University of Louisville*

[5]*Department of Acute and Tertiary Care, College of Nursing, UTHSC*

**Authors contributed equally*

a

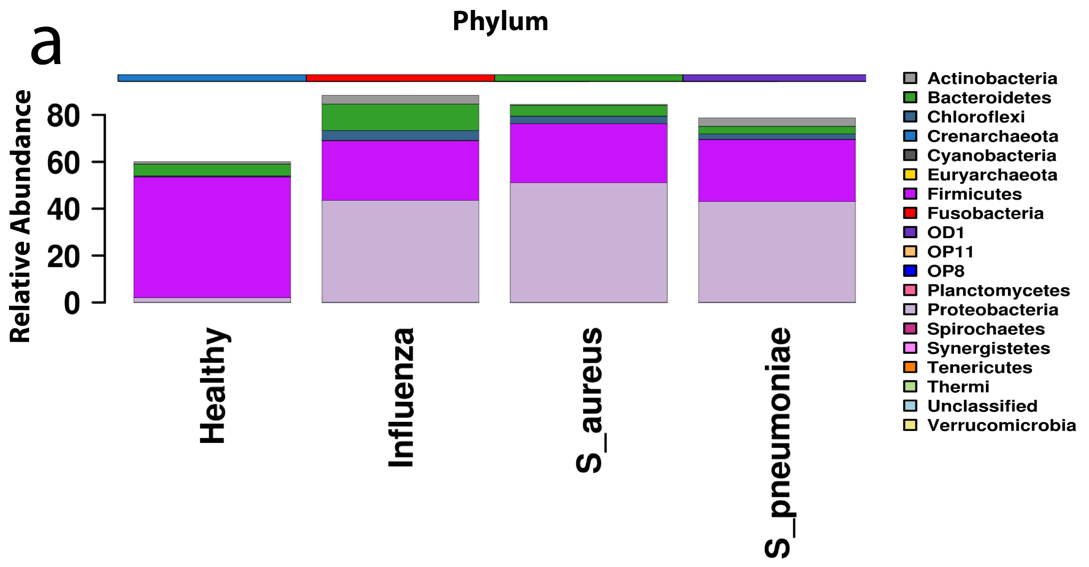

b

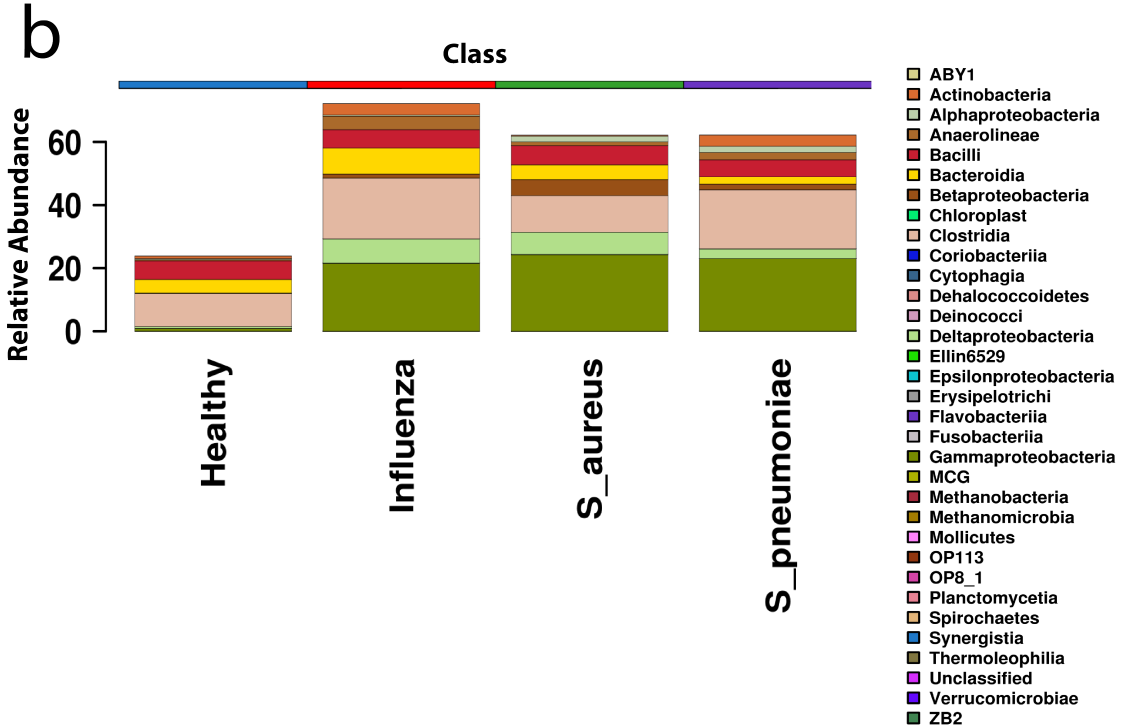

Supplemental Figure 1. Microbiome taxonomic composition by group shown at the (a) phylum and (b) class level.

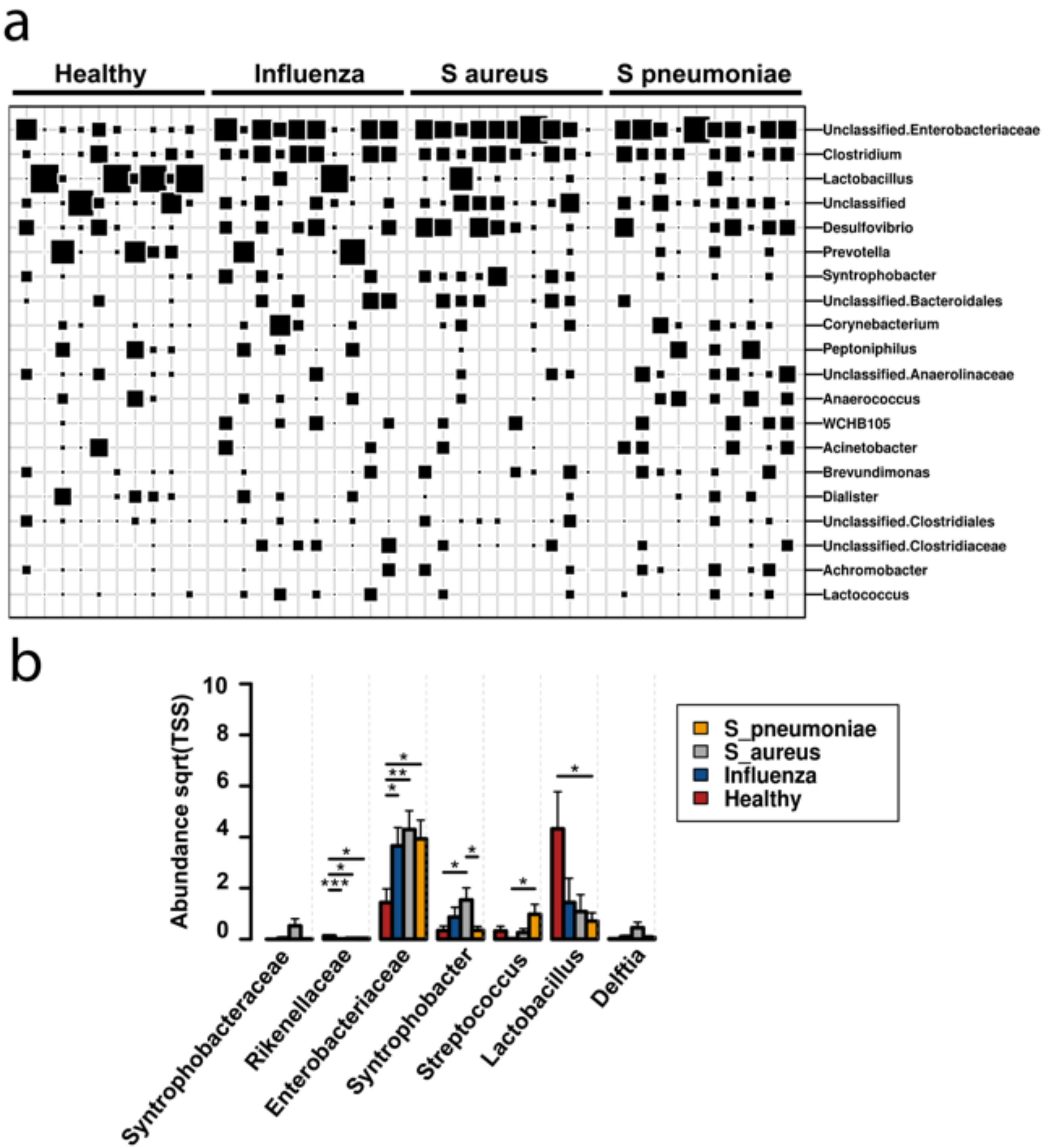

Supplemental Figure 2. (a) Box plot shows relative abundance of taxa across samples within each experimental group. (b) Anova analysis of significantly altered taxa at the family level. * P < 0.05; ** P < 0.01; *** P < 0.005.

Supplemental Figure 3. (a) Linear Discriminant Analysis of Effect Size (LefSe) of taxa enriched in healthy vs all pneumonia patients. (b) Dendrogram displaying the significantly altered taxa shown in panel a between healthy and pneumonia patients. (c) LefSe of taxa enriched in Healthy vs bacteria vs influenza patients. (d) LefSe of taxa enriched in healthy vs *S. aureus* vs *S. pneumonia* patients. (e) LefSe of taxa enriched in healthy vs influenza patients.

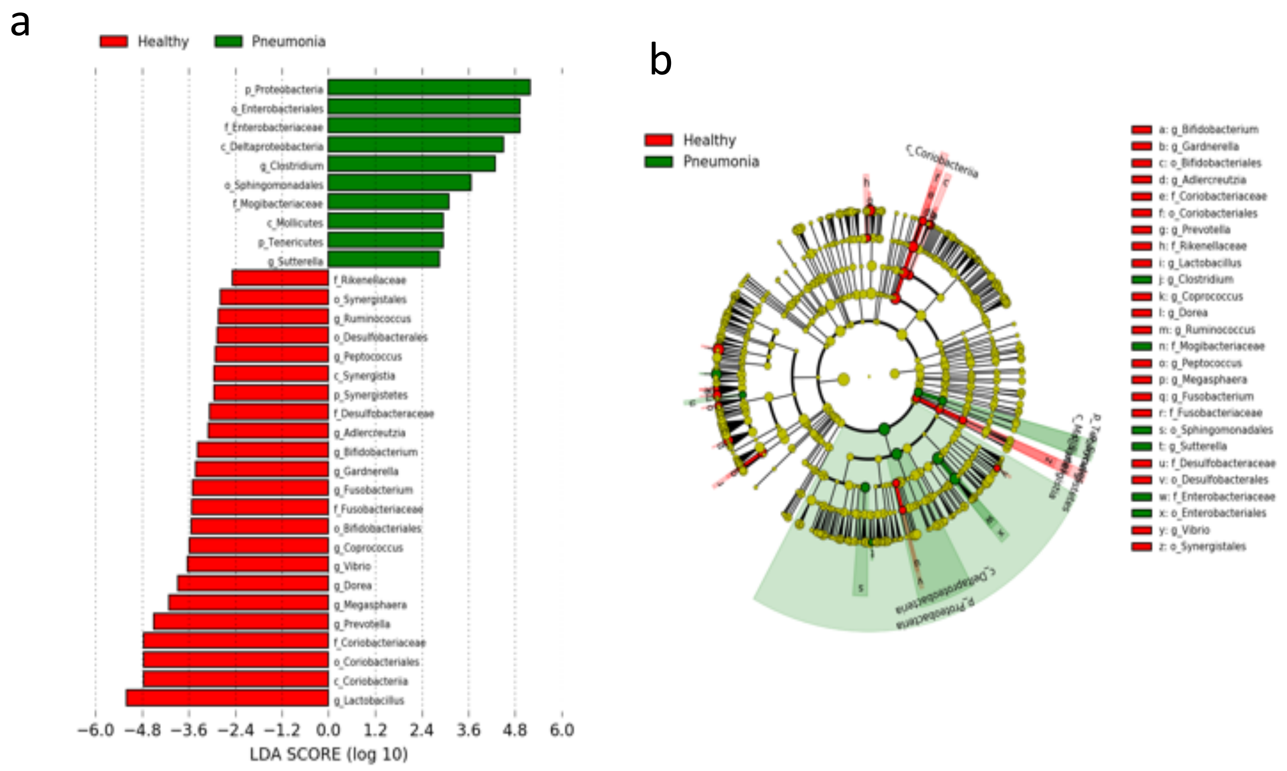

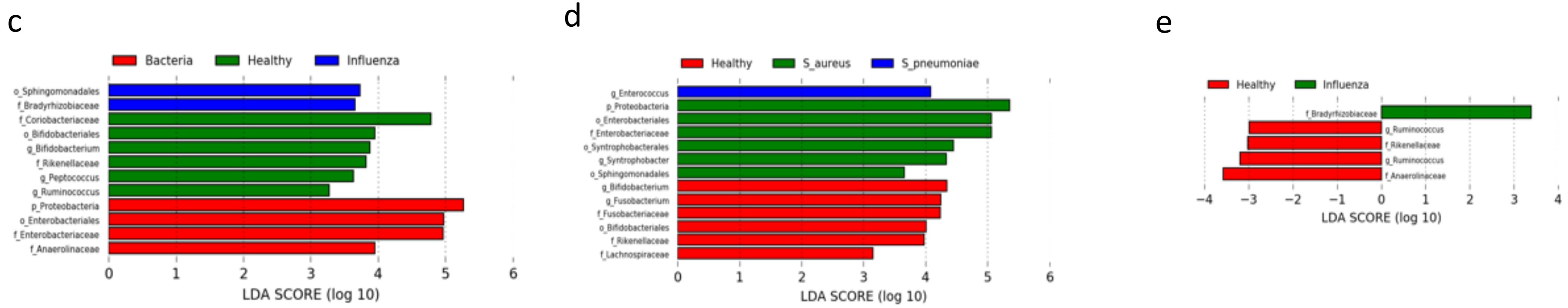

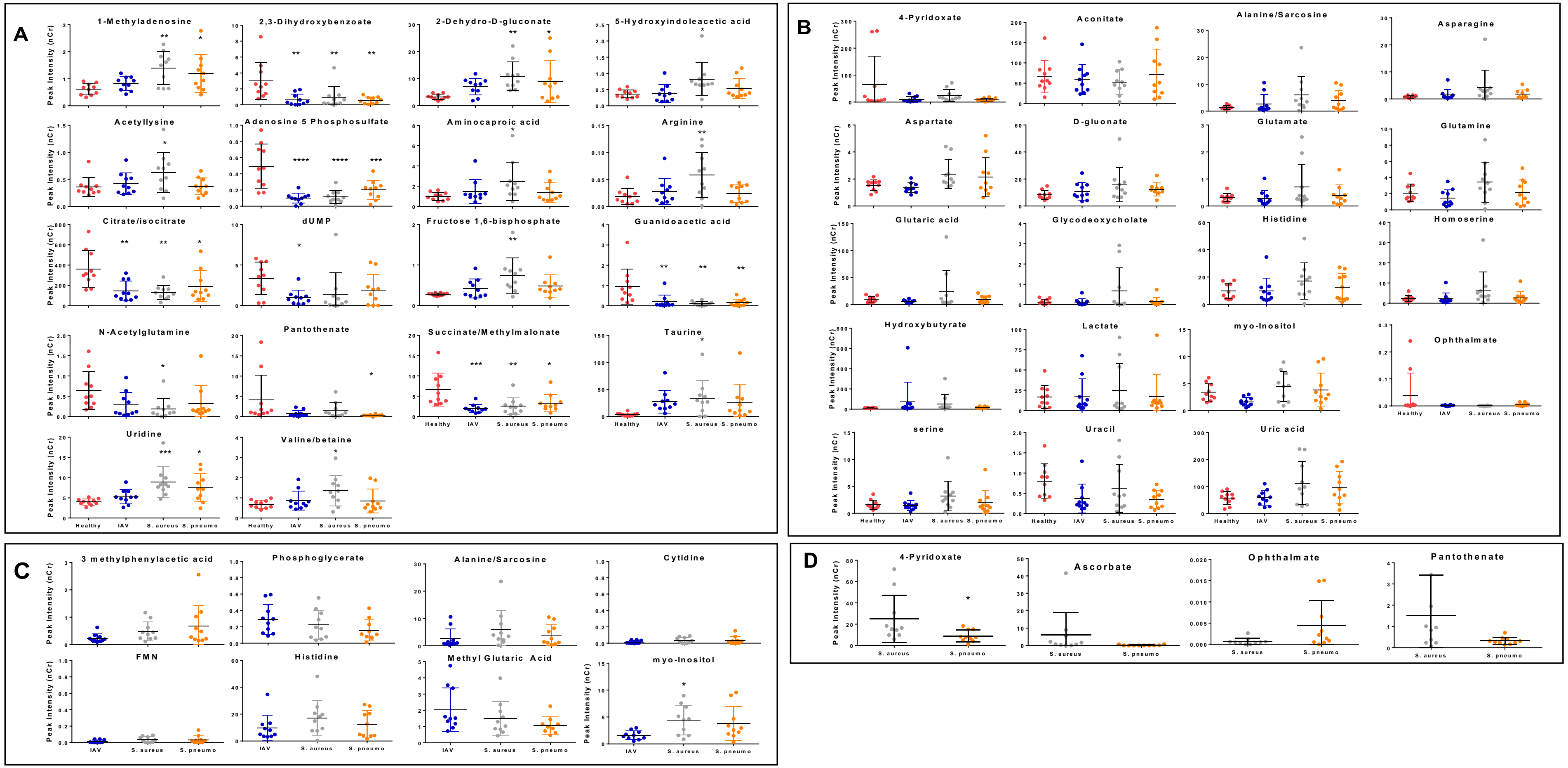

Supplemental Figure 4. Individual metabolite analysis. Samples were subjected to analysis by group (means) using one-way ANOVA with Benjamini-Hochberg post hoc correction and considered for further testing if found to have with significant differences using Tukey's honest significance test (Tukey HSD) for multiple comparisons. Each potential metabolite was then analyzed individually using ANOVA and the means compared to healthy controls using one-way ANOVA with Dunnett’s multiple comparison test. (A) Significant and (B) non-significant metabolites are shown. Infected patients were compared using one-way ANOVA with Tukey's multiple comparisons test (C). Bacterial infections were then compared using unpaired T test (D). Asterisks indicate significance as follows: p-value < 0.05 (*), p-value < 0.01(**), p-value < 0.001 (***), and p-value < 0.0001 (****).

Supp. Table 1. *Clinical characteristics of hospitalized patients due to CAP.*

Variable	Influenza	S. aureus	S. pneumoniae
n	10	10	10
Demographics			
Age (median (IQR))	54 (50-62)	61 (50-75)	61 (44-63)
Male Sex (%)	7 (70)	5 (50)	5 (50)
Caucasian (%)	8 (80)	10 (100)	8 (80)
Social and Medical History			
Neoplastic disease (%)	2 (20)	2 (20)	1 (10)
Congestive heart failure (%)	4 (40)	1 (10)	1 (10)
Stroke (%)	0 (0)	3 (30)	0 (0)
Renal disease (%)	4 (40)	3 (30)	0 (0)
Liver disease (%)	0 (0)	1 (10)	1 (10)
Diabetes (%)	3 (30)	3 (30)	3 (30)
COPD (%)	5 (50)	4 (40)	4 (40)
Human immunodeficiency virus	1 (10)	0 (0)	0 (0)
Active coronary artery disease (%)	3 (30)	1 (10)	2 (20)
Arterial hypertension (%)	8 (80)	7 (70)	7 (70)
Hyperlipidemia (%)	6 (60)	5 (50)	1 (10)
Prior myocardial infarction (%)	3 (30)	0 (0)	1 (10)
Prior PTCA/CABG (%)	3 (30)	1 (10)	1 (10)
Atrial fibrillation (%)	2 (20)	1 (10)	1 (10)
Obese (%)	6 (60)	0 (0)	5 (50)
Smoking status (%)			
Current	5 (50)	6 (60)	5 (50)
Former	5 (50)	2 (20)	2 (20)
Never	0 (00)	2 (20)	3 (30)

Physical Examination and Laboratory Findings	Influenza	S. aureus	S. pneumoniae
Heart rate (Beats/Minute)	118 (110-125)	112 (103-115)	113 (103-131)
Respiratory rate (Breaths/Minute)	28 (26-30)	27 (21-35)	26 (21-33)
Systolic blood pressure (mmHg)	122 (103-151)	109 (88-123)	102 (82-124)
Diastolic blood pressure (mmHg)	58 (49-65)	50 (43-58)	54 (42-67)
Temperature (Degrees Celsius)	38 (38-39)	38 (38-39)	38 (37-38)
Hematocrit(%)	40 (39-41)	28 (25-32)	40 (31-40)
Serum sodium (mEq/L)	135 (133-138)	129 (126-134)	135 (133-136)
Blood Urea Nitrogen (BUN) (mg/dL)	19 (13-23)	25 (18-40)	15 (13-24)
Serum bicarbonate (mEq/L)	26 (24-27)	22 (19-26)	26 (25-28)
Serum glucose (mg/dl)	112 (91-156)	149 (119-241)	124 (117-150)
PaO2/FiO2 < 250 (%)	4 (40)	3 (30)	4 (40)
Multilobar Pneumonia (%)	6 (60)	6 (60)	5 (50)
Pleural Effusion (%)	2 (20)	4 (40)	3 (30)
Severity of Disease			
Pneumonia severity index class IV/V	6 (60)	7 (70)	6 (60)
Non-invasive mechanical ventilation on day 0 (%)	0 (0)	1 (10)	1 (10)
Invasive mechanical ventilation on day 0 (%)	1 (10)	2 (20)	0 (0)
Vasopressors on day 0 (%)	0 (0)	1 (10)	0 (0)
Altered mental status on day 0 (%)	3 (30)	1 (10)	2 (20)

Clinical Course	Influenza	S. aureus	S. pneumoniae
Acute MI during hospitalization (%)	0 (0)	1 (10)	0 (0)
Need for invasive ventilation after day 0 (%)	0 (0)	2 (20)	1 (10)
Need for vasopressors after day 0 (%)	1 (10)	2 (20)	2 (20)
Time to first antibiotics (hours)	2.8 (2.3-3.8)	2.1 (1.8-3.1)	2.8 (2.0-3.5)
Time to Clinical Stability (days)	2 (2-2)	5 (2-7)	2 (2-5)
Length of Stay (days)	4 (3-4)	8 (7-13)	5 (4-9)
Outcomes			
Rehospitalization due to CAP (%)	1 (10)	0 (0)	0 (0)
Mortality during hospitalization (%)	0 (0)	0 (0)	1 (10)
Mortality at 30 days (%)	0 (0)	1 (10)	1 (10)
Mortality at 6 months (%)	0 (0)	1 (10)	1 (10)
Mortality at 1 year(%)	0 (0)	1 (10)	3 (30)

Supp. Table 2. - *Comparisons of potential metabolite biomarkers identification methods.*

Metabolite	Tukey's HSD	PLS-DA	PLS-DA VR	Lasso model	Dunnett's test	
					Pass	Fail
1-Methyladenosine			1		1	
2,3-Dihydroxybenzoate	1		1	1	1	
2-Dehydro-D-gluconate			1	1	1	
4-Pyridoxate		1	1			1
5-Hydroxyindoleacetic acid (5-HIAA)			1		1	
Acetyllysine			1		1	
Aconitate		1				1
Adenosine 5--phosphosulfate	1		1	1	1	
Alanine/Sarcosine			1			1
aminocaproic acid			1		1	
Arginine			1		1	
Asparagine			1			1
Aspartate			1			1
Citrate/isocitrate	1	1	1		1	
D-Gluconate		1				1
dUMP			1	1	1	
Fructose 1_6-bisphosphate			1		1	
Glutamate			1			1
Glutamine			1			1
glutaric acid		1				1
Glycodeoxycholate			1			1
Guanidoacetic acid	1		1	1	1	
Histidine			1			1
Homoserine/Threonine			1			1
hydroxybutyrate		1				1
Lactate		1				1
myo-Inositol			1			1
N-Acetylglutamine			1	1	1	
Ophthalmate			1			1
Pantothenate			1		1	
Serine			1			1
Succinate/Methylmalonate	1		1		1	
Taurine		1	1	1	1	
Uracil			1			1
Uric acid		1				1
Uridine	1		1		1	
Valine/betaine			1		1	

Supp. Table 3. *Errors per analysis method*

	Error Type	
	I	II
Tukey's HSD	0	12
PLS-DA	7	16
PLS-DA VCR	13	0
Lasso Model 1	0	11

Supp. Table 4. *OTUs and Metabolites selected by LASSO*

Type	Variables	Models		
		1	2	3
OTU	k_Bacteria|p_Actinobacteria|c_Actinobacteria|o_Bifidobacteriales|f_Bifidobacteriaceae|g_Bifidobacterium		X	
	k_Bacteria|p_Actinobacteria|c_Coriobacteriia|o_Coriobacteriales|f_Coriobacteriaceae|g_Adlercreutzia	X		
	k_Bacteria|p_Bacteroidetes|c_Flavobacteriia|o_Flavobacteriales|f_Weeksellaceae|g_Cloacibacterium		X	
	k_Bacteria|p_Firmicutes|c_Bacilli|o_Lactobacillales|f_Aerococcaceae|g_Facklamia		X	
	k_Bacteria|p_Firmicutes|c_Clostridia|o_Clostridiales|f_Lachnospiraceae|g_Moryella		X	
	k_Bacteria|p_Firmicutes|c_Clostridia|o_Clostridiales|f_Tissierellaceae|g_Anaerococcus			X
	k_Bacteria|p_Proteobacteria|c_Betaproteobacteria			X
	k_Bacteria|p_Proteobacteria|c_Betaproteobacteria|o_Burkholderiales			X
	k_Bacteria|p_Proteobacteria|c_Deltaproteobacteria|o_Desulfovibrionales|f_Desulfovibrionaceae		X	
	k_Bacteria|p_Proteobacteria|c_Deltaproteobacteria|o_Syntrophobacterales|f_Syntrophobacteraceae			X
	k_Bacteria|p_Proteobacteria|c_Epsilonproteobacteria|o_Campylobacterales|f_Helicobacteraceae			X
	k_Bacteria|p_Tenericutes|c_Mollicutes|o_Mycoplasmatales|f_Mycoplasmataceae|g_Ureaplasma		X	
	k_Bacteria|p_Verrucomicrobia|c_Verrucomicrobiae|o_Verrucomicrobiales|f_Verrucomicrobiaceae|g_Akkermansia			X
Cytokine	IL_18 pg/mg creatinine		X	X
	IL_15 pg/mg creatinine		X	
Metabolite	2_3_Dihydroxybenzoate	X		
	3_Phosphoglycerate		X	
	4_Pyridoxate			X
	Acetylcarnitine			X
	Adenosine 5__phosphosulfate	X		
	Arginine			X
	Ascorbate			X
	FMN		X	
	Guanidoacetic acid	X		
	methyl glutaric acid		X	
	myo_Inositol		X	
	Ophthalmate			X
	Pantothenate			X
